# Microstructure Evolution and Mechanical Properties of Thick 2219 Aluminum Alloy Welded Joints by Electron-Beam Welding

**DOI:** 10.3390/ma16217028

**Published:** 2023-11-03

**Authors:** Zhilong Chang, Minghui Huang, Xiaobo Wang, Houqin Wang, Guangda Sun, Li Zhou

**Affiliations:** 1 College of Mechanical and Electrical Engineering, Central South University, Changsha 410083, China; 2 Beijing Institute of Astronautical Systems Engineering, Beijing 100076, China; 3 State Key Laboratory of Advanced Welding and Joining, Harbin Institute of Technology, Harbin 150001, China

**Keywords:** electron beam welding, modification welding, aluminum alloy, microstructure evolution, mechanical properties

## Abstract

In this study, 2219 aluminum alloy thick plate was joined by electron beam welding. Defect-free joints with excellent surface formation were obtained. There were significant differences in the microstructure along the thickness direction of the weld zone (WZ). The upper region of the WZ was mainly striated grains, while the lower region was fine equiaxed grains. The WZ of 2219 joint is composed of α-Al and Al-Cu eutectic. Fine equiaxed grains were formed in the partially melted zone (PMZ) due to the existence of high-melting nucleation particles including Ti-Al and Ti-Zr compounds. The eutectic microstructure in the PMZ and the heat-affected zone (HAZ) presented net-like and block-shape distribution. Due to the formation of fine grains and high content of Al-Cu eutectic, the WZ showed the highest microhardness (80 HV). Therefore, the 2219 joint obtained excellent mechanical properties. The tensile strength of the 2219 joint was equal to that of the base metal (BM), but the elongation of the 2219 joint significantly decreased to 15.1%, about 67.7% of that of BM. The fracture mode of the 2219 joint presented typical ductile fracture.

## 1. Introduction

With the development of society, the rate of energy consumption has accelerated. Saving resources and developing the green economy have become a priority in current society. The concept of structural lightweight design is gaining more and more attention in the fields of vehicle transportation and aerospace [1,2]. Due to excellent properties, such as low cost, high specific strength, good fatigue strength, and corrosion resistance, aluminum alloys are widely used as an important structural material in the aerospace field. In addition, 2xxx aluminum alloys with minor Cu and Mg alloying elements display good processing properties and are mainly applied in the skins, trusses, and wing frames of airliners [3]. Therefore, it is important to achieve high-quality joints in airframe structures by advanced joining methods.

For the welding of aluminum alloy, there have been challenging weldability problems associated with aluminum alloys to overcome. Due to no solid-state phase transformations of aluminum alloy upon the cooling process, the microstructure of aluminum alloy joints is determined by the solidification process. As well as the microstructure factors, the features and defects that may contribute to the loss of properties comprise porosity, oxide inclusions, and hot cracking [4].

At present, most published papers about aluminum alloys welding are focused on friction stir welding [5,6,7], which could eliminate the fusion problems because of the solid state during joining. However, as for the complex joint geometry, friction stir welding may be difficult to apply. Fusion welding can also meet the welding requirements [8,9]. Among them, high-energy beam welding, such as laser beam welding (LBW) and electron beam welding (EBW), is recognized to obtain better joint performance and has a broad application space and potential [10,11,12]. Owing to the high-energy density [13,14] and vacuum protection, EBW could obtain high-aspect-ratio joint and suppress the formation of pores [15]. Therefore, EBW is particularly suitable for the welding of aluminum alloys.

For recent papers, microstructure and macroscopic defects in aluminum joint with EBW have been reported. Fujii et al. [16] investigated the relation between pores and bubble generation. He found that the bubbles were formed through a reaction between the molten Al and Al_2_O_3_ forming Al_2_O. Mastanaiah et al. [17] reported that the extensive liquation and segregation of Cu in the partially melted zone (PMZ) increased in the thick-plate EBW, which resulted in the failure of PMZ on the 2219Al side of the 2219/5083 joint. S.A. Hosseini et al. [11] evaluated the hot cracking sensitivity of 2024 joint by EBW and investigated the effects of heat input on the hot cracking. The conclusions showed that decreasing the heat input alleviated thermal stresses and strains improved the grain structure, hence preventing nucleation and the growth of hot cracks in the weld metal. According to Jian Wang et al. [14], the mechanical properties of EBWed joints was significantly affected by the formation of precipitated phases. The dissolution of precipitated phases caused the softening in the EBWed joint. For this reason, post-weld heat treatment to obtain precipitated phases is a feasible way to enhance the mechanical properties of the joint [9,18,19]. Fadaeifard et al. [19] pointed out that β-phase (Al_3_Mg_2_) in the weld zone of 6061Al joint precipitated along grain boundary subjected to post weld heat treatment (PWHT), improving the mechanical properties of weld zone which surpassed the base metal. In addition, some researchers pointed out that the periodic oscillation of the molten pool was an important factor to control the pore defects in the aluminum joint [13,20,21]. Therefore, excellent-quality EBWed joints can be obtained through process parameter modulation.

However, concave defects on the surface of EBWed joints have been reported in some studies [13], but the corresponding solutions to the above defect have not been discussed. In this work, the electron beam modification welding (MW) was used to improve the surface forming of 2219 joints. In addition, the microstructure evolution and mechanical properties of the joint after MW were also discussed.

## 2. Experimental

**Figure 1:**
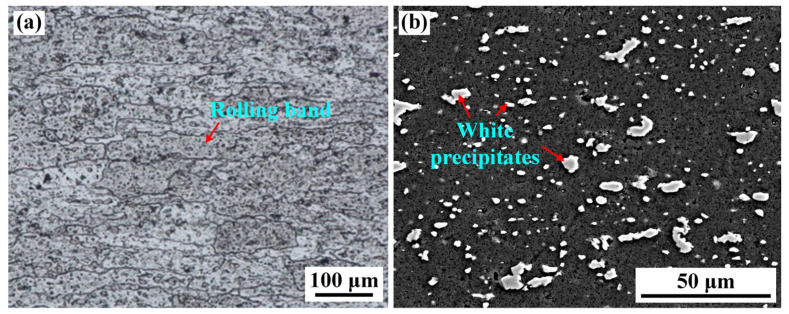
Microstructure of the BM (**a**) OM, (**b**) SEM.

The base metal (BM, 10 mm in thickness) used in this work was annealed 2219 aluminum alloy (O-state annealing process: 420 °C/12 h and furnace cooling to 250 °C). The chemical compositions of BM are shown in Table 1. Figure 1 demonstrates the microstructure of BM. The strip-like grains are distributed along the rolling direction, showing a typical rolling feature (Figure 1a). The grain size is up to 200 μm. A large number of irregular white precipitates appear in BM, as shown in Figure 1b. Based on the reported result, the white particles are enriched in Al and Cu elements, identified as θ-Al_2_Cu phase. The content of θ-Al_2_Cu precipitates is about 7.28% (area fraction).

THDW-60 large electron beam welder (Guilin Thd Technology Co., Ltd., Guilin, China) was used in this work. The direct welding (DW) and modification welding (MW) parameters are shown in Table 2. Due to the large thickness of the 2219 plate, we used a higher heat input with 120 kV accelerating voltage and 44 mA beam current. In addition, according to published literature [22,23], surface modification by electron beam could improve the surface forming of the EBWed joint. Therefore, modification welding with lower heat input was applied. The cross-section of the 2219 joint was cut perpendicular to the welding direction by EDM cutter. Metallographic sandpaper (200 grit → 400 grit → 800 grit → 1000 grit → 1500 grit → 2000 grit) and diamond polishing (1.0 μm) solution were used for rough grinding, fine grinding, and polishing. The polished metallographic specimens were etched with Keller reagent (1.5 mL HCl + 1 mL HF + 2.5 mL HNO_3_ + 95 mL H_2_O) for 60 s. The prepared specimens were analyzed using PME3 optical microscope (Olympus Corporation, Tokyo, Japan) for specimen surface formation and cross-sectional morphology. The NovaNano450 scanning electron microscope (FEI Company, Hillsboro, OR, USA) was used to analyze the microstructure, tensile fracture, and local area energy spectrum of the joints. Electron back-scatter diffraction (EBSD) analysis was carried out on samples polished with conventional mechanical polishing followed by electro-polishing with perchloric acid, ethanol, methanol, and distilled water. The EBSD camera attached to FEI Quanta scanning electron microscope (FEI Company, Hillsboro, OR, USA) and HKL Channel 5 software (Version: 5.11.20405) were used to collect and process the orientation information. The KB30S-FA microhardness tester (KB Prüftechnik, Nordsen, Germany) was used to detect the microhardness of 2219 joint along the upper, middle, and lower areas of the 2219 joint due to the large thickness of the specimen. The test was performed by 200 g load, 10 s holding time, and 0.5 mm distance between two points. Tensile tests were performed on an INSTRON-5569 universal material testing machine (Instron®, Norwood, MA, USA). Three tensile specimens for BM and joint were tested to ensure the reliability and repeatability of data.

## 3. Results and Discussion

### 3.1. Weld Forming

**Figure 2:**
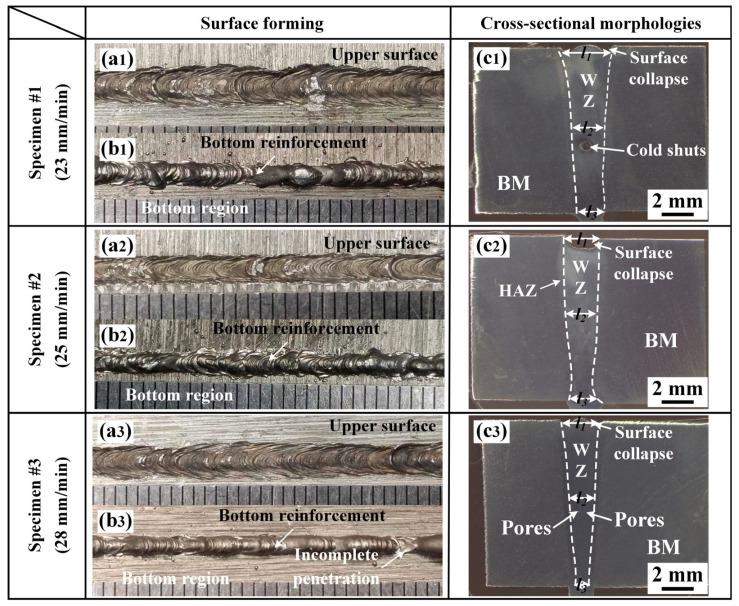
Surface forming and cross-section of the joints with welding speed of (**a1**,**b1**,**c1**) 23 mm/min, (**a2**,**b2**,**c2**) 25 mm/min and (**a3**,**b3**,**c3**) 28 mm/min.

**Figure 3:**
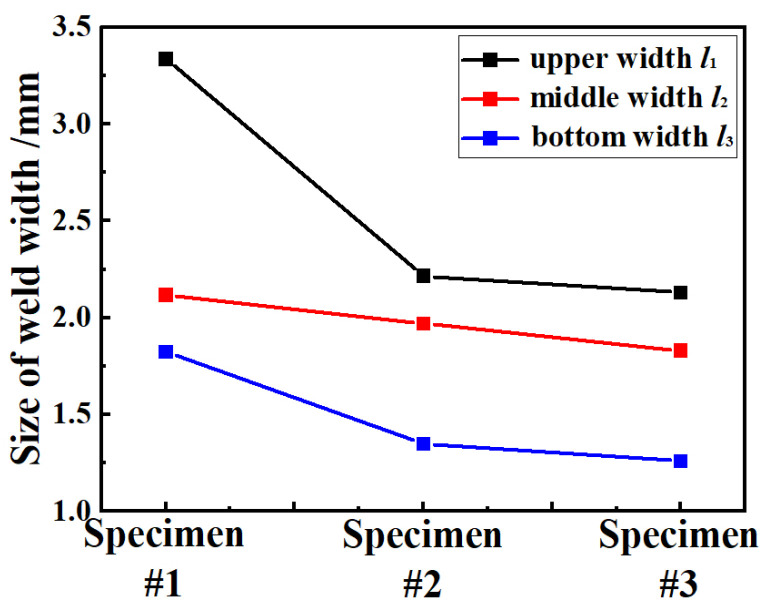
Weld width of 2219 joint with different welding speed.

The surface forming and cross-sectional morphology of the 2219 joint with different welding speeds are shown in Figure 2. With the increase in welding speed, the cross-sectional morphology of 2219 joint changed significantly. The weld widths of 2219 joints were measured by optical microscope. Three measurement values were required in each position of 2219 joint and the average results are shown in Figure 3. With the increased welding speed, the weld width of 2219 joints decreased. It is clear that several defects are formed in weld zone (WZ). However, WZ in specimen #1 produces a larger pore with a size of about 0.75 mm, which is presumed to be a possible cold shut. Cold shut is a special form of pore, causing by the obstruction of metal vapor overflow. In addition, the lower heat input in specimen #3 decreases the cooling rate of the 2219 joint, resulting in the formation of pores [24]. It also induces the partial incomplete penetration, as shown in Figure 2(b3). Based on the above analysis, the welding process of specimen #2 is thought to be the optimized welding process parameter. However, obvious surface collapse exists in the 2219 joint of specimen #2. Węglowski et al. reported that MW with defocus electron beam could improve the surface forming [22]. The secondary MW were conducted and specimen #4 was obtained.

**Figure 4:**
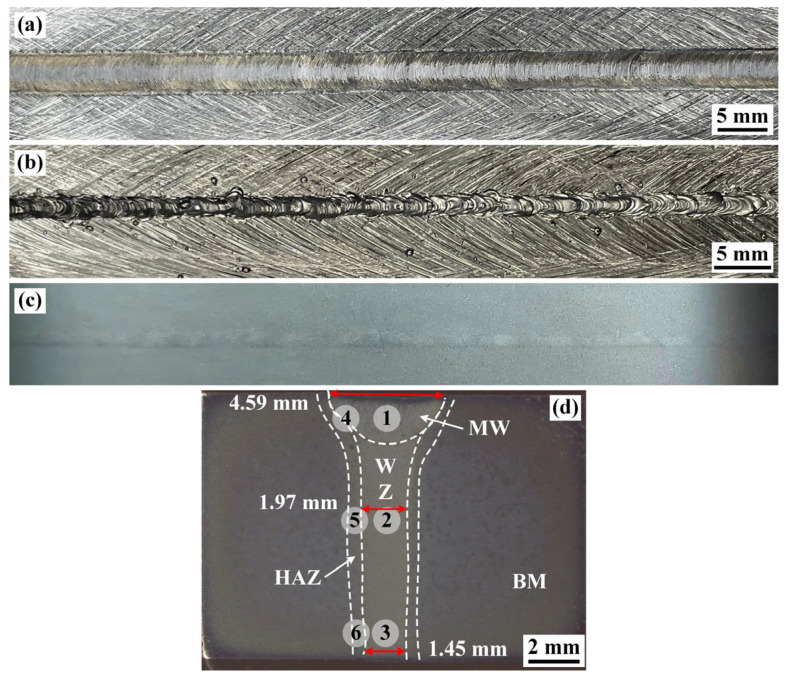
Surface formation and cross-sectional shape of specimen #4 after the secondary MW: (**a**) Top surface; (**b**) bottom surface; (**c**) X-ray inspection photograph; (**d**) cross-sectional shape of the joint.

The surface shape and cross-sectional morphology of specimen #4 are shown in Figure 4. After the secondary MW, the depth of surface collapse ranged from 0.7 mm to 0 mm, which indicates that MW can effectively improve the surface forming of 2219 joints. The defocus electron beam with larger size spot caused a significant increase in the molten alloy of upper surface. Therefore, the collapse region of the upper surface was easily filled by the molten alloy, showing a better appearance of 2219 joint. Compared with specimen #2, the weld width of specimen #4 increased from 2.22 mm to 4.59 mm after the secondary MW. The result from X-ray inspection indicates that no welding defects were found in the 2219 joint, which further verifies the optimized welding process. In addition, the 2219 joint was composed of four distinct regions, including BM, heat-affected zone (HAZ), partially melted zone (PMZ), and WZ, which were identified across the welded joint.

### 3.2. Microstructure Evolution

#### 3.2.1. Microstructure Evolution in the Thickness Direction

**Figure 5:**
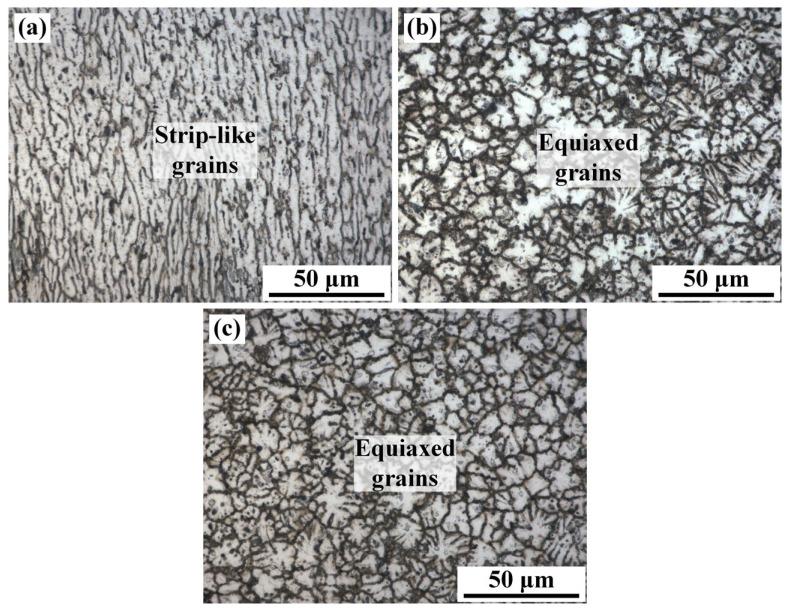
Metallographic microstructure of specimen #4: (**a**) upper, (**b**) middle, (**c**) bottom of the WZ.

Figure 5 shows the microstructure of WZ at different positions in specimen #4, which corresponds to region 1–3 in Figure 4. With the increased distance to the upper region of the 2219 joint, the grain morphology changed from strip shape to equiaxed shape, and the grain size also increased, which was due to the non-uniformity of heat input along the thickness direction. The electron beam first acted on the surface of the BM, causing the strongest thermal action of electron beam in the upper part of the WZ. Compared with the upper metal of the WZ, the middle and lower region of WZ absorbed less heat. The action of the electron beam on the middle and lower part was weakened. In addition, the contact between the bottom part and the fixture promoted the heat dissipation, which further accelerated the cooling rate. According to the solidification theory [25], the grain size and morphology were dependent on the thermal gradient (G) and the growth rate (R) in the solidification. The value of G × R, which is the same as the cooling rate, determined the grain size of the WM. According to Equation (1), the greater the distance (*h*) to the upper region of the WZ, the higher the thermal gradient. Therefore, the thermal gradient and the G × R value increased with welding depth during the welding of thick plates. Eventually, a large number of fine and equiaxed grains nucleated in the middle and bottom region of the WZ, which is similar to the results from published literature [11].

(1) $$G=2\pi k\rho c{\left(\frac{h}{Q}\right)}^{2}v{\left(T_{m}-T_{0}\right)}^{3}$$

where *k* is the thermal conductivity, *c* is the thermal capacity, *Q* is the density of the specimen, *v* is the welding speed, *h* is the thickness of the welded plate, and *T_m_* is freezing temperature, taken as ((*T_L_* + *Ts*)/2).

**Figure 6:**
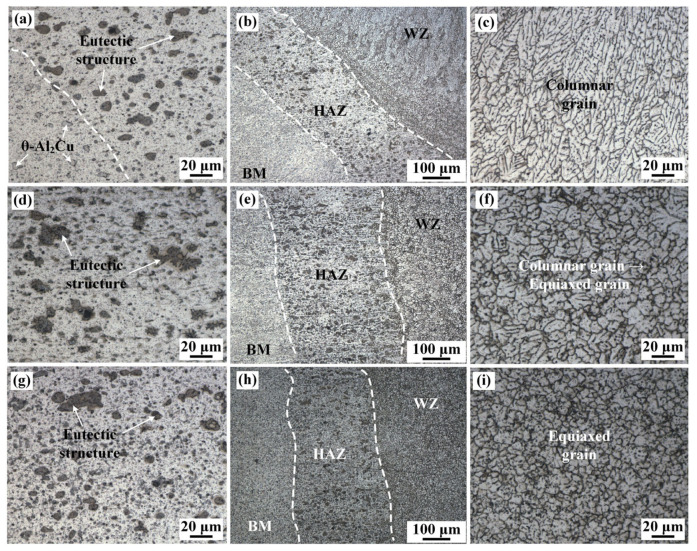
Metallographic microstructure of specimen #4: (**a**–**c**) upper, (**d**–**f**) middle, (**g**–**i**) bottom regions of 2219 joint, including the WZ (**c**,**f**,**i**), HAZ (**b**,**e**,**h**), and magnified HAZ (**a**,**b**,**g**).

Figure 6 shows the optical microstructure of the WZ, HAZ, and BM along the thickness directions of 2219 joint. With the increase in distance to the upper surface, the WZ microstructure near the fusion line in Figure 6c,f,i transformed from columnar grain to equiaxed grain, which is similar to the microstructure in Figure 5. The microstructure of HAZ is displayed in Figure 6b,e,h, corresponding to region 4, 5, and 6 in Figure 4d, respectively. The width of HAZ was between 235 and 388 μm. A handful of black precipitates was formed in HAZ and the size of precipitates was larger than that of BM, which is due to the local dissolution of θ-Al_2_Cu precipitates and the subsequent eutectic reaction.

#### 3.2.2. Phase Composition

**Figure 7:**
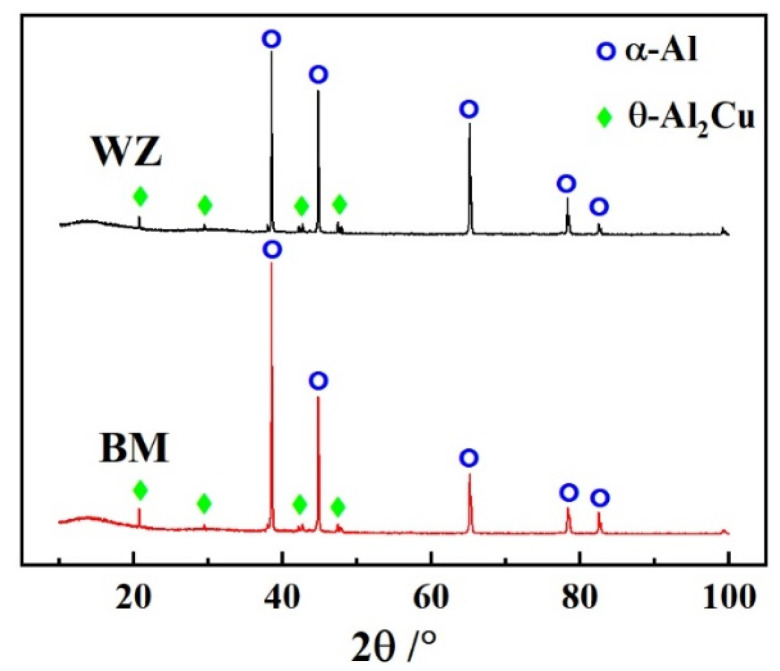
XRD results of WZ (specimen #4) and BM.

Figure 7 represents the XRD result of WZ and BM. The results indicate that WZ and BM consist of α-Al and θ-Al_2_Cu precipitate. The formation of θ-Al_2_Cu precipitate plays a strengthening role in the joints [14].

**Figure 8:**
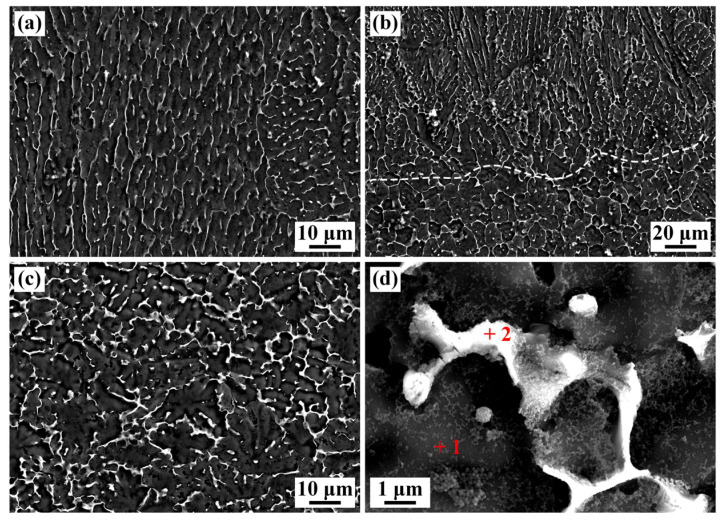
The SEM microstructure of WZ (specimen #4): (**a**) MW region; (**b**) MW transition region; (**c**) DW region; (**d**) enlarged precipitated phase in (**c**).

Figure 8 shows the SEM microstructure of the WZ with different positions. The WZ is divided into three parts: the MW region, the MW transition region, and the DW region. The MW region is mainly columnar dendrite grain, while the DW region is equiaxed grain. In addition, a large number of fine white precipitates were observed and distributed between grain boundaries (GBs) and interdendrites of WZ, which is due to the shorter elevated temperature holding time and extremely fast cooling rate in the process of EBW. As a result, fine precipitates with a content of 10.8% (area fraction) presented discrete and uniform distribution [26]. The different microstructural feature in the WZ also changed the morphologies of precipitates. Strip-like precipitates dominated in the MW region. Apart from the strip-like precipitates, some granulate precipitates were also formed in the DW region.

EDS was used to detect the phase compositions of white precipitates in the WZ and the results are shown in Table 3. Al elements were enriched in the matrix (spot 1), up to 96.4% in mass fraction. It was identified as an α-Al matrix. The content of Cu elements in white precipitates (spot 2) was about 37.7%. According to the Al-Cu phase diagram [27], the weight percentage of the Al-Cu eutectic point was 66.8% and 33.2%, which approaches the composition of spot 2 in this work. It was determined that the white precipitates were α-Al + θ-Al_2_Cu eutectics. Zhang et al. also reported similar results [28].

**Figure 9:**
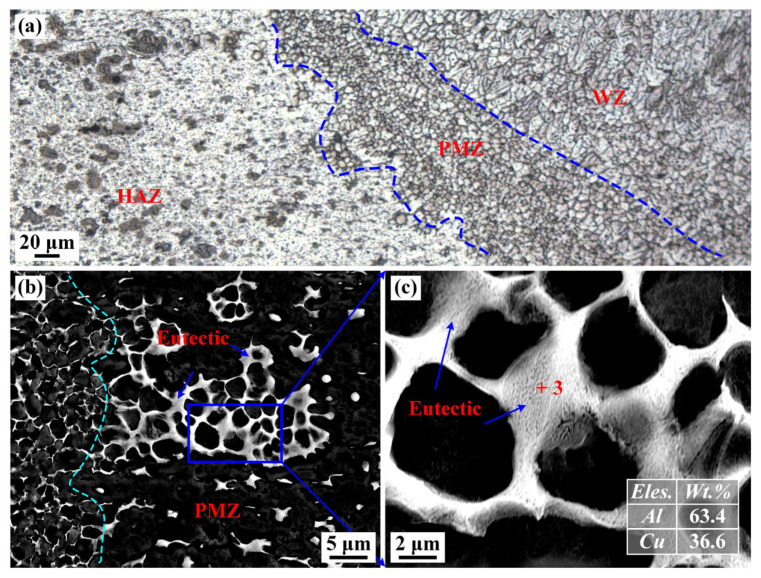
(**a**) Microstructure in PMZ (specimen #4); (**b**,**c**) enlarged SEM images of PMZ.

Figure 9 shows the microstructure of PMZ. The width of the PMZ was relatively narrow, only about 50–100 μm. Fine equiaxed grains could be observed in the PMZ, which is consistent with the obtained conclusion in the literature [29]. As shown in Table 1, 2219 aluminum alloy includes minor Ti and Zr atoms, forming Ti-Al and Ti-Zr compounds with a high melting point. These compounds could easily be nucleation particles of the melting alloy. Consequently, the grains in the WZ and PMZ were refined. In addition, some white precipitates with uneven size existed in the PMZ, which mainly distributed in GBs and interdendrites. The enlarged microstructure indicates that the white precipitates in PMZ presented a net shape, as shown in Figure 9c. As seen from the EDS results in Figure 9c, the ratio of Al to Cu in spot 3 was 1.67 (wt.%), similar to the composition of Al-Cu eutectic point. Hence, the net-like precipitates in PMZ were α-Al + θ-Al_2_Cu eutectic. Due to the closer distance to the WZ, the peak temperature in PMZ was far over the eutectic temperature (T_E_, 548.2 °C), resulting in the eutectic transformation of α-Al and θ phase. When the temperature in PMZ was lower than T_E_ during the cooling process, the net-shape Al-Cu eutectic was precipitated from the liquid alloy.

**Figure 10:**
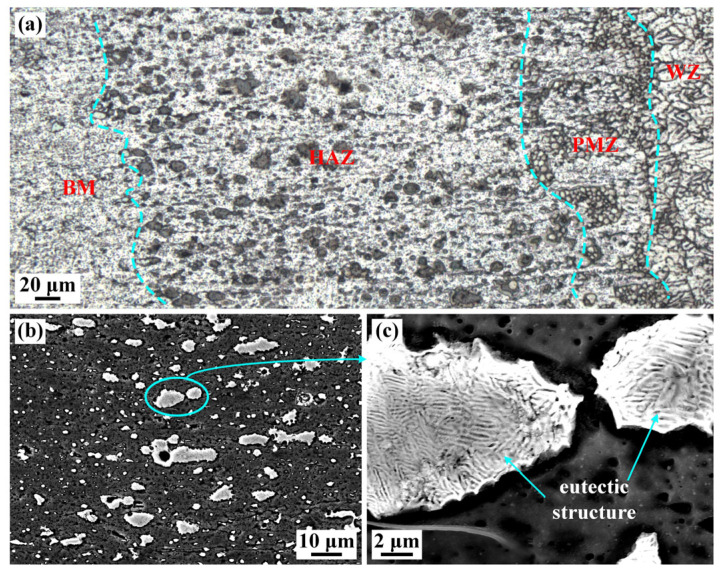
(**a**) Metallographic microstructure in the HAZ and (**b**,**c**) SEM image of the HAZ (specimen #4).

Figure 10 shows the microstructural feature of the HAZ. The white precipitates with a size of 6–11 μm presented inhomogeneous distribution, which is recognized as Al-Cu eutectic. Since the HAZ is close to the WZ, the peak temperature was higher (>550 °C). The Al_2_Cu phase underwent dissolution. The low melting point eutectic phase was infiltrated along the GBs. During the subsequent cooling process, the Al-Cu eutectic was precipitated to form block shape. Hence, the coarse block eutectic microstructure appeared in the HAZ.

#### 3.2.3. Grain Structure Evolution

**Figure 11:**
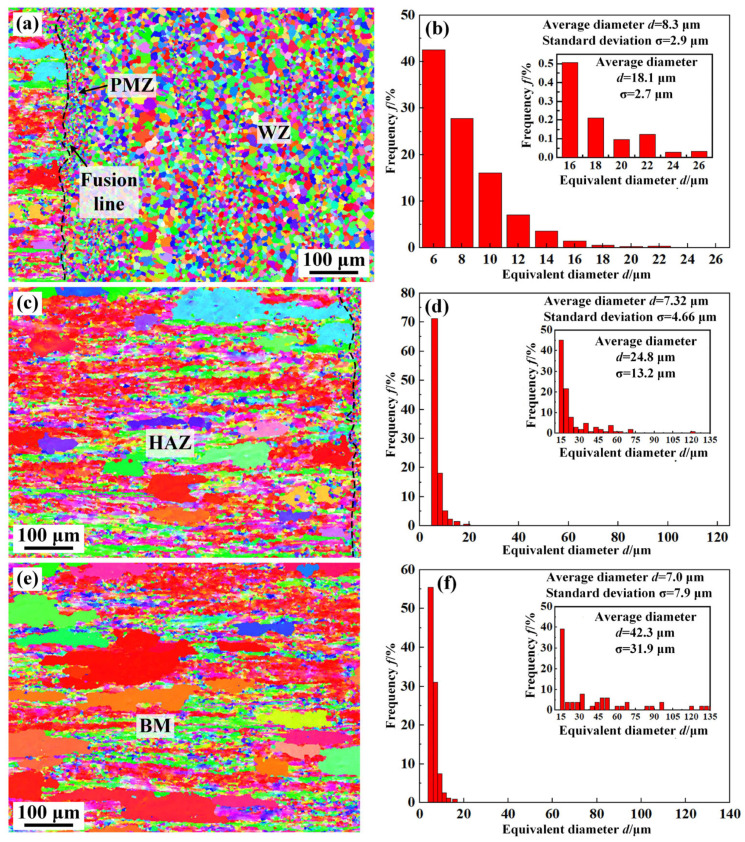
Grain morphology and statistical dimensions of the (**a**,**b**) WZ, (**c**,**d**) HAZ, (**e**,**f**) BM (specimen #4).

Figure 11 shows the morphology and size of grains in different regions of the 2219 joint. As can be seen from Figure 11, there are two types of grains in the BM and the HAZ, which are the primary coarse solidified grains and fine sub-grains. After rolling and annealing, the primary grains were partially recrystallized to form a large number of fine sub-grains with an average size of 7.0 μm in the BM. The morphologies of microstructure in the HAZ are similar to the BM. The average grain size is slightly larger than that of the BM, about 7.3 μm. The PMZ and WZ were composed of fine equiaxed grains, corresponding to the results in Figure 9a. However, the size of the grains in the WZ were larger than that of PMZ, which is relevant to the melting state. As a partially melted zone, some unmelted Al_2_Cu particles still existed in the PMZ and could easily be in the nucleation position. Thus, finer grains were formed in the PMZ. To further analyze the grain size in the different regions of the 2219 joint, only the size of primary solidified grains (>15 μm) and the results are shown in Figure 11b,d,f. A significant reduction in the size of the primary solidified grains from the BM to the WZ is observed, which implies that the mechanical properties of WZ can be dramatically improved [13].

**Figure 12:**
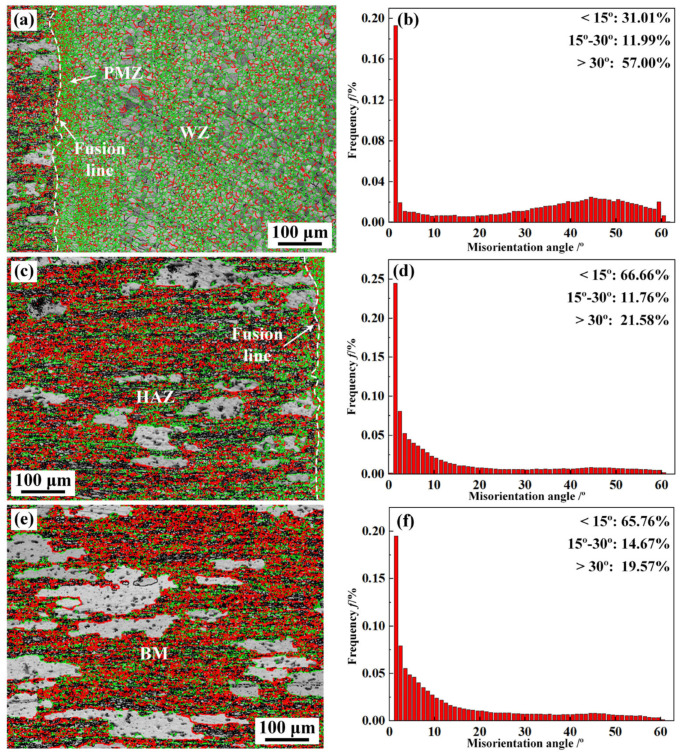
The GB characteristics and grain misorientation angle of (**a**,**b**) WZ, (**c**,**d**) HAZ, (**e**,**f**) BM (specimen #4).

Figure 12 shows the distribution of grain misorientation angle in different zones of the 2219 joint. As can be seen from Figure 12, the percentages of low-angle grain boundaries (LAGBs, <15°) and high-angle grain boundaries (HAGBs, ˃15°) in the WZ were 31.01% and 68.99%, respectively, which indicates that the WZ was dominated by HAGBs. The distribution of grain misorientation angle in BM and HAZ were similar, both dominated by LAGBs, about 65%. The BM and HAZ were rolled and underwent annealing treatment, resulting in partial recrystallization of grains and the formation of sub-grains. The grain orientation spreading (GOS) of sub-grains was generally less than 2° [30]. Consequently, the BM and the HAZ were dominated by LAGBs. In addition, Fei et al. reported that HAGBs are conducive to suppressing crack propagation [31]. The dominated HAGBs in the WZ further elevate the mechanical properties of WZ [32].

**Figure 13:**
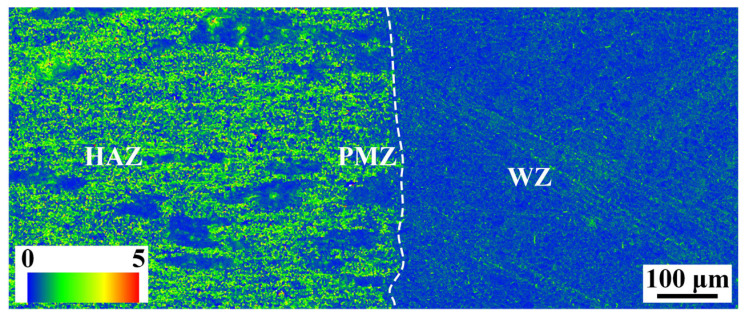
Local orientation distributions in the HAZ and WZ of 2219 joint (specimen #4).

Local orientation distributions in the PMZ, OAZ, and HAZ of the joint are shown in Figure 13. The difference of local orientation can reflect the deformation degree of the grains and the distribution of dislocation density. Compared with the WZ, the deformation in the regions of BM, HAZ, and PMZ was obvious. Among these four regions, the dislocation density of the HAZ was the highest and that of the WZ was the lowest. Before welding, the BM (2219 aluminum alloy) was subjected to 10% cold deformation and the subsequent O-state annealing heat treatment could not completely eliminate the deformation. The WZ consisted of fine grains subjected to the solidification process, resulting in the lower residual deformation. However, the peak temperature of the HAZ was very low, which basically had no effect on the residual deformation from the BM in the process of welding. As a result, the dislocation density in the HAZ was higher than that in the PMZ and WZ.

### 3.3. Mechanical Properties of 2219 Joint

**Figure 14:**
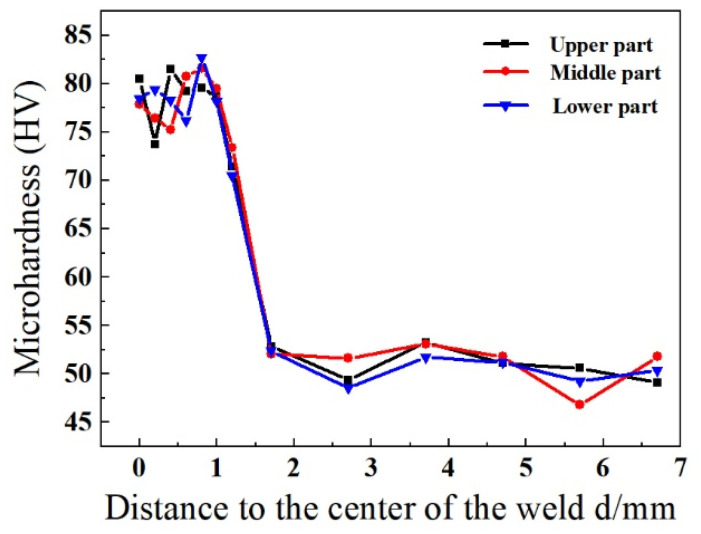
Microhardness distribution of the 2219 joint (specimen #4) with different positions.

The microhardness distribution of the 2219 joint was tested and the obtained results are demonstrated in Figure 14. The microhardness of the 2219 joint gradually decreased from the BM to the WZ. Compared with the BM, the average microhardness of the WZ was 80 HV, about 154% of that of the BM. On the one hand, the extremely high cooling rate of the WZ brought about a remarkable reduction in the grain size of the WZ. On the other hand, the content of θ-Al_2_Cu precipitates in the WZ (10.8%) was far higher than the BM (7.28%), showing an excellent strengthening effect. As a result, the WZ presented the highest microhardness.

**Figure 15:**
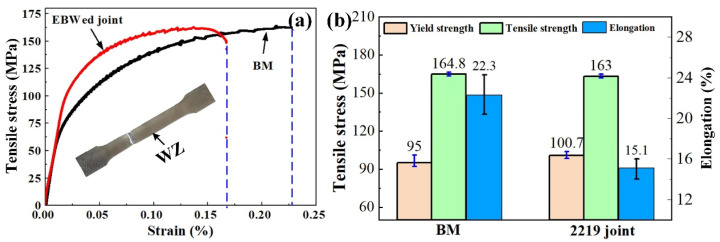
(**a**) Tensile stress–strain curve and (**b**) tensile properties of 2219 joint (specimen #4) and BM.

Figure 15 shows the tensile properties of the 2219 joint. The tensile strength of the 2219 joint was equal to that of the BM, about 163 MPa, and the 2219 joint fractured at the BM. However, the elongation of the joint was prominently reduced to 15.1%, only 67.7% of that of the BM. This was mainly due to the existence of fine grains and precipitates in the WZ and the HAZ. As seen in Figure 11, the grain size from WZ to BM decreased gradually. According to the Hall−Petch relationship described by Equation (2), the yield strength of alloy has a negative correlation with the average grain size [33]. Therefore, the tensile strength of BM was lower than that of HAZ and WZ. In addition, there were some θ-Al_2_Cu particles in the WZ and Al-Cu eutectic in the HAZ. Mastanaiah et al. found that the θ-Al_2_Cu particles precipitated from α-Al matrix destroyed the integrity and induced the initiation of cracks during the deformation process [17]. Thus, the 2219 joint showed a lower elongation.

(2) Rp0.2=Rpure+kd−12

where *R_p_*_0.2_ is the yield strength of alloy, *R_pure_* is the yield strength of pure metal, *k* is a constant, and *d* is the average grain size.

**Figure 16:**
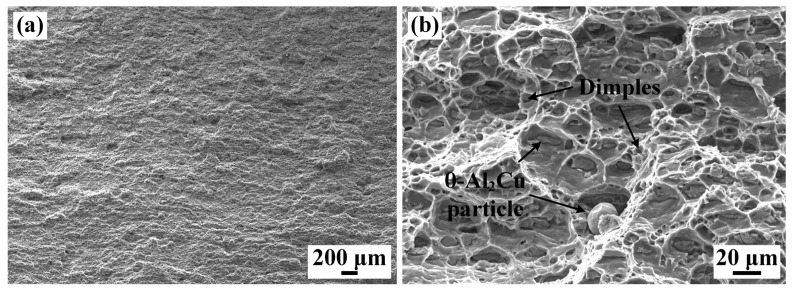
SEM images of the fractured surface of the 2219 joint (specimen #4): (**a**) low-magnification morphology; (**b**) enlarged dimples and second-phase particles.

The fracture morphology is shown in Figure 16. It can be seen that there were a large number of dimples, which was due to the nucleation, growth, and aggregation of microcavities during the tensile test. The fine second-phase particles in the dimples are clearly visible. This confirms that the fracture mode of the 2219 joint was typical ductile fracture.

## 4. Conclusions

This paper analyzed the microstructure evolution and mechanical properties of the 2219 joint. Obvious welding defects were formed in the 2219 joint with the direct EBW, which affected the mechanical properties of the 2219 joint. Thus, the MW was put forward to restrain the formation of welding defects. The main conclusions were as follows:

(1) The 2219 joint by the DW shows a poor surface forming with obvious surface collapse. MW could improve the surface forming of 2219 joint by surface remelting with lower heat input. Thus, defect-free 2219 joint is successfully achieved by EBW.
(2) The WZ shows a different microstructure along the thickness direction. With the increased distance to upper surface, the microstructure of the WZ is transformed from strip shape to equiaxed shape. The WZ is composed of α-Al and θ-Al_2_Cu precipitates, while some net-like and massive eutectic are formed in the PMZ and HAZ.
(3) Fine equiaxed grains are visible in the WZ and PMZ. Apart from the coarse solidified grains primary, the rolling and subsequent annealing treatment promote the formation of fine sub-grains resulting from partial recrystallization. The grain size of the WZ (18.1 μm) is far smaller than that of BM (42.3 μm). In addition, the WZ is dominated by HAGBs (68.99%), while the HAZ and BM are dominated by LAGBs, about 65%.
(4) The microhardness of the 2219 joint decreases gradually from the WZ to the BM. The 2219 joint fractures along BM, showing an equivalent strength of BM (163 MPa). However, the elongation of 2219 joint was only 15.1%, only 67.7% of that of the BM, related to the strengthening effect of θ-Al_2_Cu precipitates in the WZ and Al-Cu eutectic in the HAZ. The formation of dimples in the fractured surface indicates that the fracture mode of the 2219 joint presented typical ductile fracture.

## Figures and Tables

**Table 1:** Chemical composition of the BM (wt.%).

Elements	Si	Fe	Cu	Mn	Mg	Zn	Ti	V	Zr
2219-O	0.059	0.12	6.19	0.26	0.011	0.013	0.065	0.12	0.17

**Table 2:** Direct and modification welding parameters of EBW.

		Accelerating Voltage *U*/kV	Beam Current *I_b_*/mA	Focus Beam *I_f_*/mA	Welding Speed *v*/(mm·s^−1^)	Scanning Waveform	Scanning Frequency *f*/Hz	Scanning Amplitude *d*/mm
#1	DW	120	44	1945 (Surface focusing)	23	Triangular wave	1600	1.5
#2					25			
#3					28			
#4	DW	120	44	1945	25	Triangular wave	1600	1.5
	MW	120	14	1995	20	Triangular wave	150	2

**Table 3:** The EDS results of spot 1 and 2 in Figure 7 (wt.%).

	Al	Cu	Possible Phase
1	96.4	3.6	α-Al
2	62.3	37.7	α-Al+θ-Al_2_Cu eutectic

## Data Availability

The processed data required to reproduce these findings cannot be shared at this time as these data form part of an ongoing study.

## References

[1] Labus Zlatanovic D., Bergmann J.P., Balos S., Pejic D., Sovilj P., Goel S. (2022). Influence of rotational speed on the electrical and mechanical properties of the friction stir spot welded aluminium alloy sheets. Weld. World.

[2] Zhang W., Sun D., Han L., Liu D. (2014). Interfacial microstructure and mechanical property of resistance spot welded joint of high strength steel and aluminium alloy with 4047 AlSi12 interlayer. Mater. Des..

[3] Dursun T., Soutis C. (2014). Recent developments in advanced aircraft aluminium alloys. Mater. Des..

[4] Totten G.E., MacKenzie D.S. (2003). Handbook of Aluminum: Physical Metallurgy and Processes.

[5] Jata K.V., Semiatin S.L. (2000). Continuous dynamic recrystallization during friction stir welding of high strength aluminum alloys. Scr. Mater..

[6] Wang S., Wei X., Xu J., Hong J., Song X., Yu C., Chen J., Chen X., Lu H. (2020). Strengthening and toughening mechanisms in refilled friction stir spot welding of AA2014 aluminum alloy reinforced by graphene nanosheets. Mater. Des..

[7] Yu P., Wu C., Shi L. (2021). Analysis and characterization of dynamic recrystallization and grain structure evolution in friction stir welding of aluminum plates. Acta Mater..

[8] Kermanidis A.T., Zervaki A.D., Haidemenopoulos G.N., Pantelakis S.G. (2010). Effects of temper condition and corrosion on the fatigue performance of a laser-welded Al–Cu–Mg–Ag (2139) alloy. Mater. Des..

[9] Zhu Z.Y., Deng C.Y., Wang Y., Yang Z.W., Ding J.K., Wang D.P. (2015). Effect of post weld heat treatment on the microstructure and corrosion behavior of AA2219 aluminum alloy joints welded by variable polarity tungsten inert gas welding. Mater. Des..

[10] Chen G., Yin Q., Zhang G., Zhang B. (2020). Underlying causes of poor mechanical properties of aluminum-lithium alloy electron beam welded joints. J. Manuf. Process..

[11] Hosseini S.A., Abdollah-zadeh A., Naffakh-Moosavy H., Mehri A. (2019). Elimination of hot cracking in the electron beam welding of AA2024-T351 by controlling the welding speed and heat input. J. Manuf. Process..

[12] Koteswara Rao S.R., Madhusudhan Reddy G., Srinivasa Rao K., Kamaraj M., Prasad Rao K. (2005). Reasons for superior mechanical and corrosion properties of 2219 aluminum alloy electron beam welds. Mater. Charact..

[13] Chen G., Liu J., Shu X., Gu H., Zhang B., Feng J. (2019). Beam scanning effect on properties optimization of thick-plate 2A12 aluminum alloy electron-beam welding joints. Mater. Sci. Eng. A.

[14] Wang J., Liu Z., Bai S., Cao J., Zhao J., Luo L., Li J. (2021). Microstructure evolution and mechanical properties of the electron-beam welded joints of cast Al–Cu–Mg–Ag alloy. Mater. Sci. Eng. A.

[15] Huang J.C., Chen S.C., Lee M.F., Shen Y.D. (1996). Joining Efficiency of Superplastic 8090 Al-Li Thin Sheets Using Electron or Laser Beam Welding. Mater. Sci. Forum.

[16] Fujii H., Umakoshi H., Aoki Y., Nogi K. (2004). Bubble formation in aluminium alloy during electron beam welding. J. Mater. Process. Technol..

[17] Mastanaiah P., Reddy G.M., Bhattacharya A., Kapil A., Sharma A. (2022). Unveiling Liquation and Segregation Induced Failure Mechanism in Thick Dissimilar Aluminum Alloy Electron-Beam Welds. Metals.

[18] Malarvizhi S., Raghukandan K., Viswanathan N. (2007). Effect of post weld aging treatment on tensile properties of electron beam welded AA2219 aluminum alloy. Int. J. Adv. Manuf. Technol..

[19] Fadaeifard F., Matori K.A., Garavi F., Al-Falahi M., Sarrigani G.V. (2016). Effect of post weld heat treatment on microstructure and mechanical properties of gas tungsten arc welded AA6061-T6 alloy. Trans. Nonferrous Met. Soc. China.

[20] Wang X., Chen H., Liu H. (2014). Investigation of the relationships of process parameters, molten pool geometry and shear strength in laser transmission welding of polyethylene terephthalate and polypropylene. Mater. Des..

[21] Yang Z., Fang Y., He J. (2020). Numerical Investigation on Molten Pool Dynamics and Defect Formation in Electron Beam Welding of Aluminum Alloy. J. Mater. Eng. Perform..

[22] Węglowski M.S., Błacha S., Phillips A. (2016). Electron beam welding–Techniques and trends–Review. Vacuum.

[23] Nahmany M., Hooper Z., Stern A., Geanta V., Voiculescu I. (2016). AlxCrFeCoNi high-entropy alloys: Surface modification by electron beam bead-on-plate melting. Metallogr. Microstruc..

[24] Chen G., Liu J., Shu X., Gu H., Zhang B. (2019). Numerical simulation of keyhole morphology and molten pool flow behavior in aluminum alloy electron-beam welding. Int. J. Heat Mass Transf..

[25] David S.A., Babu S.S., Vitek J.M. (2003). Welding: Solidification and microstructure. JOM.

[26] Li Y., Zhao Y., Wang J., Zhan X. (2021). Effect of laser power on the grain morphology and microhardness of dual laser-beam bilateral synchronous welded 2219 aluminium alloy T-joint. Sci. Technol. Weld. Join..

[27] Zobac O., Kroupa A., Zemanova A., Richter K.W. (2019). Experimental description of the Al-Cu binary phase diagram. Metall. Mater. Trans. A.

[28] Zhang C., Zhao Y., Liu D., Niu F., Ma G., Wu D. (2023). Effect of pulsed laser frequency on microstructure and mechanical properties of 2219 aluminum alloy welded joints. Opt. Laser Technol..

[29] Kang Y., Zhan X., Qi C., Shi L., Wang Q. (2019). Grain growth and texture evolution of weld seam during solidification in laser beam deep penetration welding of 2219 aluminum alloy. Mater. Res. Express.

[30] Ayad A., Allain-Bonasso N., Rouag N., Wagner F. (2012). Grain Orientation Spread Values in if Steels after Plastic Deformation and Recrystallization. Mater. Sci. Forum.

[31] Fei Z., Pan Z., Cuiuri D., Li H., Wu B., Ding D., Su L., Gazder A.A. (2018). Investigation into the viability of K-TIG for joining armour grade quenched and tempered steel. J. Manuf. Process..

[32] Wan Z., Wang Q., Zhao Y., Zhao T., Shan J., Meng D., Song J., Wu A., Wang G. (2022). Improvement in tensile properties of 2219-T8 aluminum alloy TIG welding joint by PMZ local properties and stress distribution. Mater. Sci. Eng. A.

[33] Zhang D., Wu A., Yue Z., Shan J., Wan Z., Wang G., Song J., Zhang Z., Liu X. (2020). Microstructural evolution and its effect on mechanical properties in different regions of 2219-C10S aluminum alloy TIG-welded joint. Trans. Nonferrous Met. Soc. China.

